# Maternal genetics influences fetal neurodevelopment and postnatal autism spectrum disorder-like phenotype by modulating in-utero immunosuppression

**DOI:** 10.1038/s41398-021-01472-x

**Published:** 2021-06-05

**Authors:** Ritika Jaini, Matthew R. Wolf, Qi Yu, Alexander T. King, Thomas W. Frazier, Charis Eng

**Affiliations:** 1 Genomic Medicine Institute, Lerner Research Institute, Cleveland Clinic, Cleveland, OH 44195 USA; 2 Department of Molecular Medicine, Cleveland Clinic Lerner College of Medicine, Case Western Reserve University, Cleveland, OH 44106 USA; 3 Department of Psychology, John Carroll University, University Heights, Cleveland, OH 44118 USA; 4 Autism Speaks, Cleveland, OH 44131 USA; 5 Department of Genetics and Genome Sciences, Case Western Reserve University School of Medicine, Cleveland, OH 44106 USA

**Keywords:** Autism spectrum disorders, Genetics

## Abstract

Genetic studies in ASD have mostly focused on the proband, with no clear understanding of parental genetic contributions to fetal neurodevelopment. Among parental etiological factors, perinatal maternal inflammation secondary to autoimmunity, infections, and toxins is associated with ASD. However, the inherent impact of maternal genetics on in-utero inflammation and fetal neurodevelopment in the absence of strong external inflammatory exposures is not known. We used the *Pten*^*WT/m3m4*^ mouse model for ASD to demonstrate the impact of maternal genetics on the penetrance of ASD-like phenotypes in the offspring. *Pten*^*WT/m3m4*^ (Mom^m3m4^) or *Pten*^*WT/WT*^ (Mom^WT^) females, their offspring, and placental interface were analyzed for inflammatory markers, gene expression, and cellular phenotypes at E17.5. Postnatal behavior was tested by comparing pups from Mom^m3m4^ vs. Mom^WT^. Mothers of the *Pten*^*WT/m3m4*^ genotype (Mom^m3m4^) showed inadequate induction of IL-10 mediated immunosuppression during pregnancy. Low IL-10 in the mother was directly correlated with decreased complement expression in the fetal liver. Fetuses from Mom^m3m4^ had increased breakdown of the blood–brain–barrier, neuronal loss, and lack of glial cell maturation during in-utero stages. This impact of maternal genotype translated to a postnatal increase in the risk of newborn mortality, visible macrocephaly and ASD-like repetitive and social behaviors. Depending on maternal genotype, non-predisposed (wildtype) offspring showed ASD-like phenotypes, and phenotypic penetrance was decreased in predisposed pups from Mom^WT^. Our study introduces the concept that maternal genetics alone, without any added external inflammatory insults, can modulate fetal neurodevelopment and ASD-related phenotypes in the offspring via alteration of IL-10 mediated materno-fetal immunosuppression.

## Introduction

Autism spectrum disorder (ASD) affects 1 in 54 children in the United States and imposes major health, societal and economic burdens^1^. The etiology of ASD is multifactorial and complex. Genetic contributions to the etiology of ASD are well established with high heritability of the disorder^2,3^ including concordance in monozygotic twins^4,5^. In spite of strong evidence for a genetic etiology, approximately 80% of ASD cannot be explained by monogenic mutations or copy number variants (CNVs)^6–9^. Even in monogenic ASD, penetrance is almost never complete^10–13^. Therefore, the risk architecture of ASD is thought to involve additive effects of multiple genetic mutations/single-nucleotide polymorphisms (SNPs) or CNVs as well as environmental factors, mostly of low penetrance that act in combination to overcome the threshold of clinical phenotype development.

ASD genetics has been predominantly focused on the proband in order to identify specific pathological pathways responsible for the neurological and behavioral phenotypes^10^. Few studies focus on parental genetics in ASD, and to date, have failed to identify significant associations with ASD in the offspring. Parental SNPs/CNVs highlighted in some studies are often located in the vicinity of, or within, ASD-associated genes^14–16^.

Increased risk of ASD in mothers with autoimmunity^17–19^, high disease concordance in siblings^20,21^ as well as increased incidence of ASD on perinatal exposure to pathogens or toxins^22–25^ may all converge on fetal exposure to maternal inflammation as the causative factor (reviewed in refs. ^26,27^). Increasing numbers of studies suggest an in-utero gestational etiology for ASD^28,29^. Although human studies are mostly associative, murine studies have established the role of maternal inflammation in ASD by experimental injection of strong inflammatory mediators, such as IgG, LPS, or Poly I:C during pregnancy, isolated from other confounding factors^30–34^. Maternal genetics may be an important modulator of this in-utero inflammation. Few immune response-related genes have been suggested to confer a high risk of having offspring with ASD^35–37^. However, the inherent impact of maternal genetics on ASD pathogenesis during in-utero neurodevelopment, in the absence of strong pro-inflammatory insults or ongoing inflammation is not understood. Moreover, the processes by which maternal genetics may modulate the penetrance of high-risk monogenic ASD genes in genetically predisposed offspring have not been demonstrated. Here we aim to demonstrate the inherent role of maternal genetics predisposing to inflammation, on in-utero neurodevelopment using a *Pten* mutant murine model for ASD.

*PTEN* is ranked as a category-1 (i.e., high-confidence) ASD-associated gene (SFARI database^11,38^. *PTEN* germline mutations account for as much as 2% of all ASD and 17% of macrocephalic-ASD^39^. The biological impact of PTEN function on neurodevelopment is reflected in the fact that 95% of patients with germline *PTEN* mutations have macrocephaly caused mainly by megalencephaly, and clear white matter abnormalities in the brain^40,41^. With an approximately 23% penetrance of ASD, *PTEN* germline mutations provide a good genetic model system to study the additional impact of environmental and genetic modifiers that help overcome the threshold for phenotype development. We used our *Pten* mutant murine model for ASD (*Pten*^*m3m4*^), to elucidate the impact of maternal genetics on the development and modulation of PTEN-ASD like phenotype in genetically high-risk offspring.

## Materials and methods

### Mice

All experiments were performed on the *Pten*^*WT/m3m4*^ knock-in mutant model generated on the C57Bl/6 background as described previously^42,43^. *Pten*^*WT/m3m4*^ germline mutations restrict Pten expression to the cytoplasm, resulting in a constitutive decrease in total Pten protein compared to *Pten*^*WT/WT*^. *Pten*^*m3m4/m3m4*^ homozygous mutations are embryonic lethal. All *Pten*^*WT/m3m4*^ females used for breeding were derived from *Pten*^*WT/WT*^ dams. Breeding pairs were set at 7–8 weeks of age for both genders. Mice underwent gestation in similarly controlled colony conditions till E17.5 or full term as per the experimental design.

### Animal ethics statement

All procedures were approved by the Cleveland Clinic’s Institutional Animal Care and Use Committee under protocol numbers 2018-1952 and 2017-1879 and guided by the Principles of Laboratory Animal Care formulated by the National Society for Medical Research.

### Behavior analysis

Three chamber sociability (*n* = 10), distance traveled (*n* = 9), and marble burying (*n* = 10) tests were performed as described previously^42,44^.

### Measurement of macrocephaly

Macrocephaly was visually assessed at ages P2–P40 by an investigator blinded to the maternal/pup genotype. Brains were weighed immediately post-dissection at P40. Coronal brain sections, in the same plane anterior to the hippocampus, were measured for length (L) and breadth (B) using Image J software. Area was calculated assuming an ellipse as *π*(*L*/2 × *B*).

### Multiplex bead-based cytokine assays

Serum (1:2 dilution) and uterine fluid (no dilution) were tested using the mouse high sensitivity T-cell magnetic bead panel and the mouse cytokine/chemokine bead panel as per manufacturer’s instructions (Millipore, Billerica, MA).

### Enzyme-linked immunosorbent assay (ELISA)

An antiphospholipid antibody, TGFβ2, and GDF-15 ELISAs were performed on maternal sera, uterine fluid or fetal liver at gestational day E17.5 according to manufacturer instructions (Supplementary Table 1). Absorbance was measured at 450 nm on a SynergyMx Microplate reader (BioTek, Winooski, VT).

### Immunohistochemistry/immunofluorescence

Immunohistochemistry (IHC) staining was performed as previously described for frozen sections^45^. Primary and secondary (HRP or florescent labeled) antibodies were acquired commercially (Supplementary Table 1). Images were acquired on a Leica DM4 B LED microscope using a DFC7000 T camera, and LAS X Software. Fluorescent confocal images were acquired on a Leica multi-photon confocal microscope at 630× with 3× digital zoom. Digital image analysis was performed using ImageJ (v1.52k; National Institutes of Health, Bethesda, MD). Length, area, and integrated density of microglia and albumin were calculated by drawing regions of interest in different fields per biological replicate. DAB quantifications and integrated density calculations were performed as before^45^.

### Western blotting

Quantification for Pten was performed as previously described^45^.

### Quantitative reverse transcription-polymerase chain reaction (qRT-PCR)

RNA was extracted using the standard miRNEasy protocol (Qiagen, Germantown, MD). SYBR Green qRT-PCR was performed using commercially available primers (Genecopoeia, Rockville, MD) as previously described^45^.

### Gene expression panel analysis

Total, 20 ng/µl RNA was analyzed on the Nanostring nCounter using the Murine PanCancer Immune Profiling Panel or the Murine Glia Panel. Analysis for differential gene expression was performed using the nSolver advanced software v2.0 (Nanostring Technologies, Seattle, WA).

### RNA sequencing

Mouse fetal whole brain RNA libraries were prepared using the TruSeq Standard Total RNA w/Ribo Zero Gold kit (Illumina, San Diego, CA)., Paired end 150 bp sequencing was performed on the Hi-Seq platform (Illumina). RNA Sequencing bioinformatics were conducted at the Quantitative Health Sciences department at the Lerner Research Institute. Transcripts were QC’d and reads mapping to a preset index of known cDNA transcripts were counted using the Salmon quantification tool. DESeq2 was used for differential gene expression analysis. Functional pathways highlighted by differentially expressed genes were elucidated using Ingenuity Pathway Analysis (Qiagen).

### Statistical analyses

All experiments were performed with sample sizes per group indicated in their respective results section. All analyses included different biological and technical replicates as specified tested in the same (ELISAs) or two or more separate (IHC) experimental setups. The sample size was determined based on our previous studies utilizing the Pten^m3m4^ model^42,46^. All studies except visible evaluation of macrocephaly were conducted in a non-blinded manner with sample utilization being randomized. All center statistics are depicted as means with error bars showing standard deviation. Statistical significance was determined by performing one-tailed or two-tailed Student’s *t* tests as indicated. Significance was determined at a *p* value of <0.05. Variance in fetal complement expression by maternal IL-10 was quantified by *R*^2^ values.

## Results

### Offspring of mothers with the *Pten*^*WT/m3m4*^ genotype show increased ASD-like features compared to those from *Pten*^*WT/WT*^ mothers

**Fig. 1:**
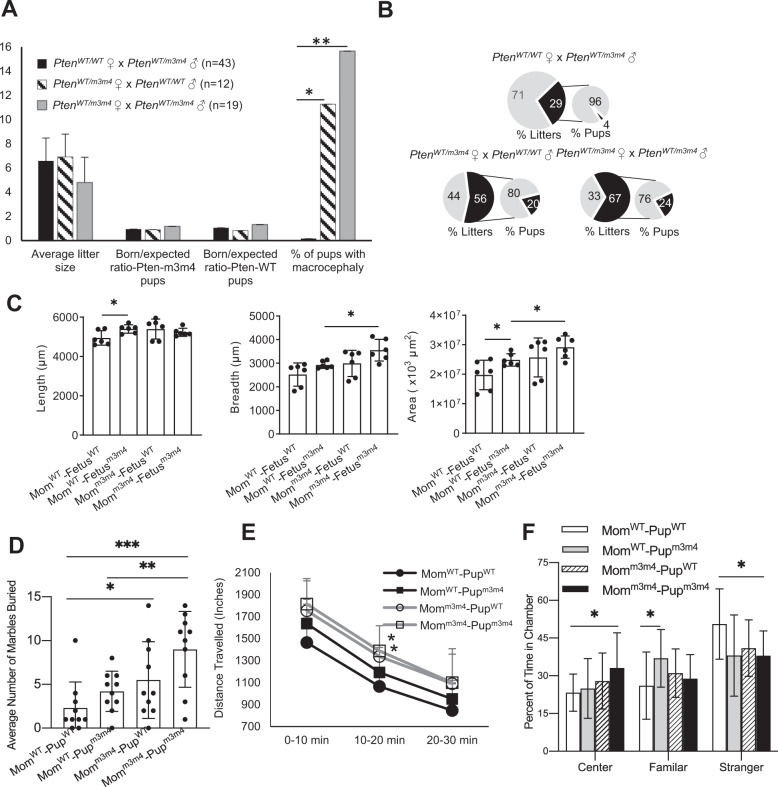
Pups of *Pten*^*WT/m3m4*^ mothers have higher postnatal mortality, macrocephaly, and ASD-like behavior compared to *Pten*^*WT/WT*^ mothers. Postnatal comparison of litters from *Pten*^*WT/m3m4*^ vs. *Pten*^*WT/WT*^ mothers showing **A** incidence of macrocephaly (one-tailed *t* test with unequal variance), **B** incidence of mortality. Light grey = % surviving. Black = % dead. **C** Quantification of macrocephaly by measurements of coronal brain sections of pups of both genotypes derived from Mom^m3m4^ vs. those from Mom^WT^ (two sections each from *n* = 3 pups/group); length (l), breadth (b), and area (calculated as $\pi\left( {\frac{l}{2}xb} \right)$, **D** marble-burying behavior analysis of pups derived from *Pten*^*WT/m3m4*^ mothers (Mom^m3m4^–Pup^WT^ and Mom^m3m4^–Pup^m3m4^) vs. *Pten*^*WT/WT*^ mothers (Mom^WT^–Pup^WT^ and Mom^WT^–Pup^m3m4^). **E** Distance traveled in three chambers. **F** Three chamber sociability behavior analysis. One-tailed *t* test; *p < 0.05; **p < 0.01; ***p < 0.001.

To interrogate the impact of maternal genetics on the incidence and severity of an ASD-like phenotype in the offspring, *Pten*^*WT/m3m4*^ females were bred to *Pten*^*WT/WT*^ males or *Pten*^*WT/WT*^ females were bred to *Pten*^*WT/m3m4*^ males to compare pups of a heterozygous mutant mother (Mom^m3m4^) vs. pups of a homozygous wildtype mother (Mom^WT^). Average litter sizes from *Pten*^*WT/WT*^ females X *Pten*^*WT/m3m4*^ males and *Pten*^*WT/m3m4*^ females X *Pten*^*WT/WT*^ males were similar. In contrast, a significant decrease in litter size was observed when *Pten*^*WT/m3m4*^ females X *Pten*^*WT/m3m4*^ males, most likely due to 100% loss of *Pten*^*m3m4/m3m4*^ homozygous mutant fetuses in-utero around E15.5. For this reason, *Pten*^*WT/m3m4*^ female X *Pten*^*WT/m3m4*^ male crosses were not compared further in the study for the maternal inflammatory state, due to additional inflammatory insults from resorption of homozygous mutant fetuses in-utero. The number of *Pten*^*WT/WT*^ (Pup^WT^) and *Pten*^*WT/m3m4*^ (Pup^m3m4^) pups born were as expected per Mendelian ratios indicating no genotype preference or in-utero mortality differences between the two maternal or offspring genotypes (Fig. 1A). However, postnatally, *Pten*^*WT/m3m4*^ mothers lost more pups between P0 (birth) and P8. Litters of Moms^m3m4^ had postnatal mortality of 20–24% of their pups in 56–67% of all litters compared to only 4% pup death in 29% of litters from Mom^WT^ (Chi^2^ = 66.66, d*f* = 1, *p* = 0.001, Fig. 1B).

Litters were examined postnatally for obviously visible macrocephaly (i.e., extreme increase in head size). *Pten*^*WT/m3m4*^ mothers (Mom^m3m4^) had significantly more pups with visible macrocephaly compared to none (*n* = 43) in litters from *Pten*^*WT/WT*^ mothers (*Pten*^*WT/m3m4*^ females X *Pten*^*WT/WT*^ males, 11%, *n* = 12, *p* = 0.01; *Pten*^*WT/m3m4*^ females X *Pten*^*WT/m3m4*^ males, 16%, *n* = 19, *p* = 0.008, Fig. 1A). Macrocephaly was quantified by measuring coronal brain sections. Interestingly, Pups^m3m4^ from Mom^m3m4^ had significantly increased brain breadth and area compared to all pups from Mom^WT^ reflecting the cortical expansion evident visibly (Fig. 1C; *n* = 2 sections/3 mice/group; length, *p* = NS; breadth, *p* = 0.01; area, *p* = 0.03). Pups^m3m4^ had increased brain size compared to Pups^WT^ only in Mom^WT^ (length, *p* = 0.02; breadth, *p* = NS; area, *p* = 0.04). In contrast, Pups^WT^ and Pups^m3m4^ from Mom^m3m4^ showed similar brain size measurements. However, the brain weight of Pups^m3m4^ was higher than Pups^WT^ only when derived from Mom^m3m4^ (Fig. S1A).

Two core ASD-like behaviors, i.e., proclivity towards repetitive behavior and sociability were compared between *Pten*^*WT/WT*^ and *Pten*^*WT/m3m4*^ pups derived from either *Pten*^*WT/WT*^ mothers (designated Mom^WT^–Pup^WT^ and Mom^WT^–Pup^m3m4^, respectively) or *Pten*^*WT/m3m4*^ mothers (Mom^m3m4^–Pup^WT^ and Mom^m3m4^–Pup^m3m4^) at age P40. Behavior differences were observed in both male (*n* = 10/group; Fig. 1D–F) and female pups (*n* = 9 per group; Fig. S1B–D). Pup^WT^ and Pup^m3m4^ males born to Mom^m3m4^ displayed significantly more marble burying and total distance traveled (measures of repetitive behavior; Mom^m3m4^–Pup^WT^, *p* = 0.01 and Mom^m3m4^–Pup^m3m4^, *p* = 0.002) compared to Mom^WT^–Pup^WT^. Mom^WT^–Pup^m3m4^ showed no significant differences in marbles buried or distance traveled compared to their wildtype littermates (Mom^WT^–Pup^WT^), in spite of their high-risk genotype. Interestingly, Pup^WT^ from Mom^m3m4^ showed a significant increase in marble-burying behavior compared to Pup^WT^ from Mom^WT^. The number of marbles buried and distance traveled by the pups was governed by maternal genotype followed by pup *Pten* genotype, such that Mom^WT^–Pup^WT^ (average marbles buried per 30 min = 2.3) < Mom^WT^–Pup^m3m4^ (4.2 marbles) < Mom^m3m4^–Pup^WT^ (5.5 marbles, *p* = 0.03) < Mom^m3m4^–Pup^m3m4^ (9 marbles; *p* = 0.0003) (Fig. 1D, E). Pup^m3m4^ derived from Mom^m3m4^ showed significantly increased marble burying compared to Pup^m3m4^ from Mom^WT^, but were interestingly very similar in marble-burying behavior to Pup^WT^ derived from Mom^m3m4^ in spite of the pup’s wildtype status. Female mice followed the same trend, although the combined effect of maternal and pup genotype on the severity of behavior was more exaggerated in the males (Fig. S1B, C).

Three-chamber sociability tests showed a decreased preference for the stranger in all groups compared to Mom^WT^–Pup^WT^ (*p* = 0.04 for Pup^m3m4^ from either mom; *p* = 0.057 for Mom^m3m4^–Pup^WT^). Interestingly, Pup^WT^ from Mom^m3m4^ showed similar sociability to Pups^m3m4^ from both maternal types. Showing aversion to the stranger, Pups^m3m4^ from Mom^m3m4^ tended to spend more time in the empty center chamber away from all animate objects (*p* = 0.04), whereas Pup^m3m4^ from Mom^WT^ preferred the familiar mouse compared to Mom^WT^–Pup^WT^ (*p* = 0.036). Females did not show a significant preference for the familiar mouse or empty chamber (Fig. S1D). Among females, only Mom^m3m4^–Pup^m3m4^ showed reduced preference to the stranger, reflecting the overall lower severity of ASD-like behavior in females.

These data collectively indicate that maternal genotype can influence neuronal abnormalities in the offspring, reflected by increased macrocephaly, postnatal mortality, and ASD-like behavior. Even wildtype offspring not genetically predisposed to neurodevelopmental disorders can be influenced by maternal genotype to develop ASD-like behavior, albeit less severe than littermates carrying the mutant alleles. In the same vein, expected neurodevelopmental phenotypes in high-risk mutant offspring (Pup^m3m4^) were seen to be modulated to milder signs under the influence of a “benign” or “non-predisposing” maternal genotype (Mom^WT^).

### Moms^m3m4^ lack induction of an IL-10-mediated immunosuppressive state during pregnancy directly correlating with decreased expression of complement in the fetus

**Fig. 2:**
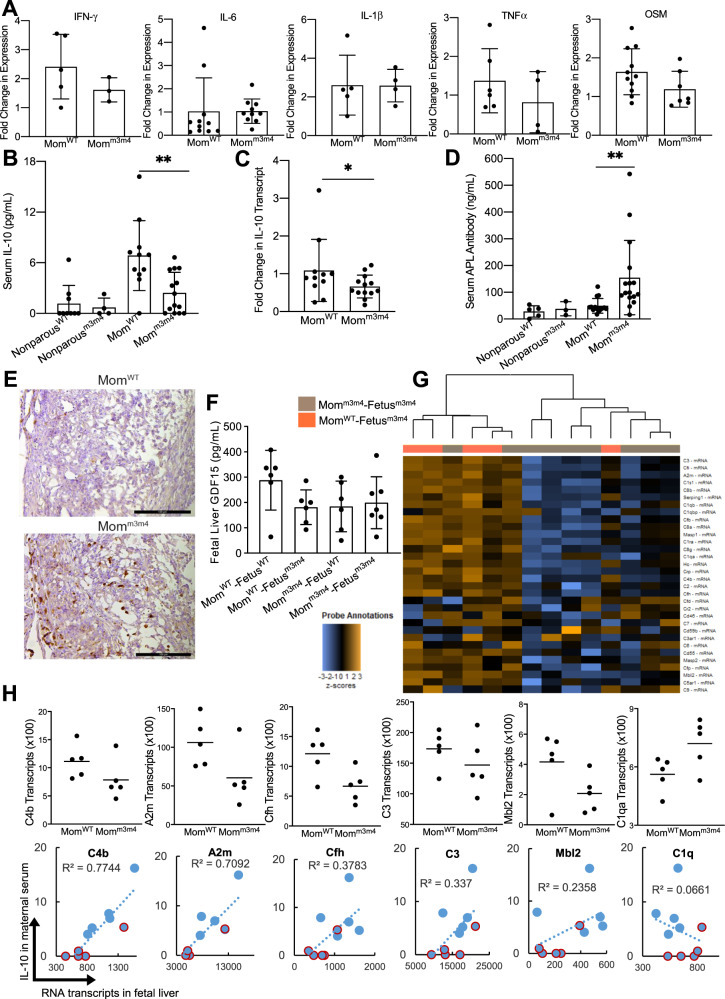
Moms^m3m4^ lack induction of an IL-10 mediated immunosuppressive state during pregnancy that correlates with decreased expression of complement in the fetus. **A** Quantitative real-time PCR for expression of pro-inflammatory cytokines in the spleen of Mom^m3m4^ vs. Mom^WT^. **B** Multiplex ELISA quantification of IL-10 in serum of pregnant females compared to non-parous females (two-tailed *t* test, **p < 0.01). **C** q-RT-PCR based fold change in IL-10 transcript in the spleen of Mom^m3m4^ vs. Mom^WT^ (one-tailed *t* test, *p < 0.01). **D** ELISA quantification for anti-phospholipid antibodies in serum of mothers (one-tailed *t* test, **p < 0.01). **E** IHC for FcγR expression in E14.5 placental decidua. Scale bars = 100 µm. **F** ELISA for GDF-15 in fetal livers at E17.5. **G** Heat map of complement pathway gene expression showing unsupervised clustering of Fetuses^m3m4^ based on their maternal genotype (Mom^m3m4^: brown vs. Mom^WT^: orange). **H** (Top panel) Nanostring analysis showing average transcript counts/mother (normalized to a housekeeping gene panel) for complement genes in fetal livers (each data point depicts the average of two fetuses/mother, *n* = 5 mothers/group). (Lower panel) correlation of complement gene expression in the fetal liver with maternal serum IL-10 levels. Blue dots: Mom^WT^, blue dots with a red outline: Mom^m3m4^.

To define what constitutes a “predisposing” maternal genotype, we first interrogated the currently most well-recognized parental factor associated with ASD-like behavior, i.e., the maternal inflammatory state. Our recent studies show that naïve, nonparous *Pten*^*WT/m3m4*^ mice do not have ongoing inflammation or autoimmunity but are predisposed to higher immune reactivity only upon stimulation^45^. To analyze the immunological status of *Pten*^*WT/m3m4*^ mice in a state of pregnancy, cytokine expression/levels were tested in the spleen of pregnant females at E17.5 by qRT-PCR. No differences in proinflammatory cytokine transcript expression were observed between Mom^m3m4^ and Mom^WT^ indicating a lack of ongoing inflammation (IFNγ, *n* = 5; IL-6, *n* = 10; IL-1β, *n* = 4; TNFα, *n* = 6; OSM, *n* = 7; Fig. 2A). In addition, testing for IL-17a showed no transcripts in the spleen from either genotype. Multiplex serum ELISA for G-CSF, GM-CSF, IFNγ, IL-1α, IL-1β, IL-2, IL-4, IL-5, IL-6, IL-10, LIF, IL-15, IL-17, KC, MCP-1, M-CSF, MIP-2, TNF-α, also showed no differences between Mom^m3m4^ and Mom^WT^, except in IL-10 levels (Fig. 2B).

Since mid-gestation pregnancy is considered to be an immunosuppressive state^47–50^, anti-inflammatory cytokines (IL-10, TGFβ1, and TGFβ2) in maternal serum were also tested by ELISA. Serum IL-10 levels were increased in pregnant females (*n* = 14) compared to nonparous females (*n* = 4) reconfirming the induction of IL-10-mediated immunosuppression during pregnancy (Fig. 2B). However, Moms^m3m4^ showed significantly decreased serum IL-10 levels (*n* = 14), as well as IL-10 transcripts (*n* = 13, *p* = 0.047) in the spleen compared to Moms^WT^ (*n* = 10, Fig. 2B, C). The majority of Moms^m3m4^ had low (<4 pg/ml) serum IL-10 (71.4% Moms^m3m4^ vs. 9% Moms^WT^, range 0–6.6 pg/ml vs. 0–16.2 pg/ml, *p* = 0.002), indicating a possible failure of induction of immunosuppression during pregnancy. As expected, a similar induction of TGFβ2 was seen in pregnant females compared to nonparous females. However, TGFβ1 and TGFβ2 levels were not significantly different between Moms^m3m4^ and Moms^WT^ (Fig. S2A, B). Increased anti-phospholipid antibodies were found in the serum of Moms^m3m4^ at E17.5 (*n* = 15, *p* = 0.003; Fig. 2D) along with increased FcγR expression in the placental decidua (maternal-derived tissue) only at E14.5 (Fig. 2E). FcγR expression was absent in the placental decidua at E17.5 in mothers of both genotypes (*n* = 3, Fig. S2C).

To assess the impact of a failure to immunosuppress on the fetus, we first tested pro-inflammatory cytokine levels in the uterine fluid which is predominantly fetus-derived. No significant differences in pro-inflammatory cytokine expression were observed in Mom^m3m4^–Fetus^m3m4^ compared to Mom^WT^–Fetus^m3m4^ (Fig. S2D). Pten protein expression was similar in mutation-carrying fetuses from either Mom genotype (Fig. S2E). GDF-15, primarily derived from the placenta is thought to be a stress sensor and inducer of maternal–fetal immuno-tolerance. Decreased GDF-15 levels have been correlated with preeclampsia and loss of pregnancy^51–53^. Lower GDF-15 levels were found in fetal livers of all Mom–Fetus combinations except Mom^WT^–Fetus^WT^ (Fig. 2F; mean 288.1 pg/ml vs. approx. 181 pg/ml in other groups, *n* = 6, *p* = 0.08), albeit not statistically significant due to the recognized variation in GDF-15 levels. This indicates possible stress signaling in Fetuses^m3m4^ from both maternal genotypes and interestingly also in Fetuses^WT^ from Mom^m3m4^. Pan-cancer immune profiling of fetal livers, the primary hematopoietic organ during fetal stages, differentially clustered complement pathway gene expression in Mom^m3m4^–Fetus^m3m4^ (*n* = 9) vs. Mom^WT^–Fetus^m3m4^ (*n* = 5), indicating substantial differences dictated primarily by their maternal genetic environments (Fig. 2G). In-depth analysis revealed almost two-fold lower complement (C4b, A2m, Cfh, C3, and Mbl2) expression in the liver of Fetus^m3m4^ from Moms^m3m4^ compared to Fetus^m3m4^ from Moms^WT^ (Fig. 2H upper panel). More importantly, lower expression of complement proteins in the fetal liver was directly correlated with low maternal serum IL-10 levels (Fig. 2H lower panel), especially C4b and A2m (*R*^2^ = 0.77 and 0.71, respectively). Only C1q was not significantly different between fetuses from different maternal genotypes or correlated with maternal IL-10 levels.

These data show that maternal genetics that merely predisposes to inflammation can impact maternal–fetal immune tolerance during pregnancy and alter fetal physiology. More importantly, this alteration of fetal processes can happen in the absence of strong external pro-inflammatory insults or ongoing maternal autoimmunity, and merely as a result of inadequate immunosuppression during the pregnancy.

### Fetus^m3m4^ from Mom^m3m4^ show increased breakdown of the blood–brain–barrier, compared to Fetus^m3m4^ from Mom^WT^

**Fig. 3:**
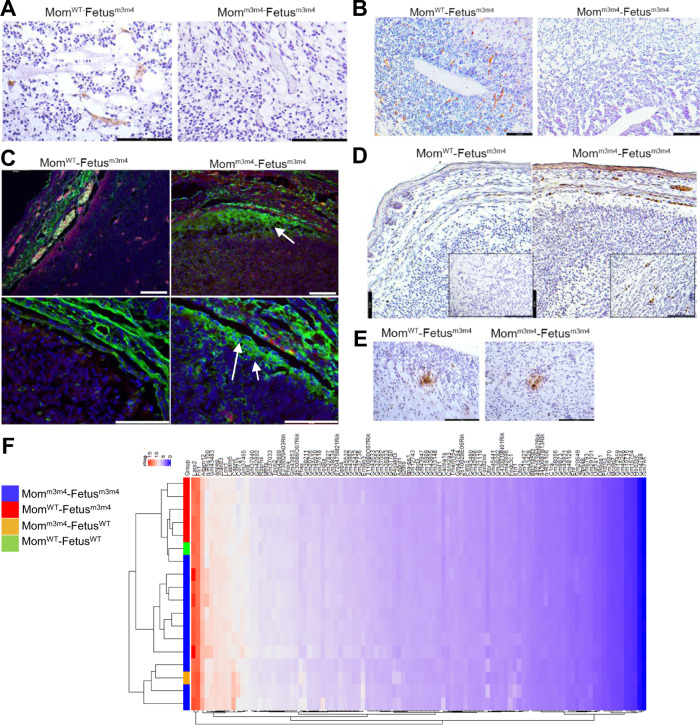
Breakdown of the blood–brain–barrier in fetuses undergoing gestation in Mom^m3m4^. IHC for **A** Glut-1 (representative of *n* = 4 mice/group) and **B** CD31 (representative of *n* = 4 mice/group) BBB markers in fetuses from Mom^m3m4^ and Mom^WT^. **C** IF staining for albumin (representative of *n* = 6 mice/group). Arrows show albumin leakage into the brain parenchyma and perivascular areas. *Lower panels* show higher magnification images. **D** IHC for complement protein C4 (representative of *n* = 6 mice/group) in blood vessels and brain parenchyma (shown in high magnification inserts). **E** IHC for complement protein C3 in the brain of fetuses derived from either maternal genotype (representative of *n* = 2 mice/group). Scale bars = 100 µm. **F** Heat map of fetal brain gene expression at E17.5 showing unsupervised differential clustering of Mom^m3m4^ fetuses (blue and orange) from Mom^WT^ fetuses (red and green).

We next investigated the differential impact of maternal genetics on the developing fetal brain. Significantly decreased expression of markers of the blood–brain–barrier (BBB), namely, glucose transporter 1 (Glut-1) and endothelial cell integrity, i.e., platelet endothelial cell adhesion molecule (PECAM-1 or CD31) was observed in Mom^m3m4^–Fetus^m3m4^ compared to Mom^WT^–Fetus^m3m4^ (*n* = 4, Figs. 3A, B and S3A, B). Decreased CD31 expression was not a result of reduced angiogenesis in Mom^m3m4^–Fetus^m3m4^ as evident by equal expression of CD31 in extra central nervous system (CNS) tissue (Fig. S2F). Significantly increased albumin leakage was seen in perivascular spaces in the brain of Mom^m3m4^–Fetus^m3m4^ indicating a break-down of the BBB in-utero at E17.5 (*n* = 6, Figs. 3C and S3C). In contrast to the decrease in expression of C4b transcripts in the liver (Fig. 2H), the brain of Mom^m3m4^–Fetus^m3m4^ showed increased C4 deposits in blood vessels as well as the brain parenchyma (*n* = 6, Fig. 3D). Complement C3 deposits however were not different between Mom^m3m4^–Fetus^m3m4^ and Mom^WT^–Fetus^m3m4^ (*n* = 2, Fig. 3E).

Comparative bulk RNA sequencing analysis of fetal brains differentially clustered Mom^m3m4^–Fetus^m3m4^ (*n* = 12) separately from Mom^WT^–Fetus^m3m4^ (*n* = 6, Fig. 3F), indicating significant transcription differences between Fetuses^m3m4^ just based on their maternal genotypes. Ninety-one protein-coding genes showed ≥2-fold expression difference (*P* corrected < 0.03) in Mom^m3m4^–Fetus^m3m4^ compared to Mom^WT^–Fetus^m3m4^ (Supplementary Table 2). Among these, the top most differentially expressed gene was that coding for Claudin 22 (12.6× linear fold-change, *p* = 0.02). Claudin 22 is involved in calcium-independent, cell-cell adhesion via the plasma membrane, further supporting a breakdown of the BBB. Interestingly, only 1/91 differentially expressed genes were complement-related indicating that the observed increase in complement deposits in the brain is likely peripherally derived and not a result of altered complement gene expression within the brain.

Collectively, these data show that maternal genetic contributions that promote a lack of immunosuppression during pregnancy can lead to a breakdown of the BBB in the fetus.

### Fetuses of Mom^m3m4^ have increased neuronal loss, lack of astrocyte and microglia maturation compared to fetuses from Mom^WT^

**Fig. 4:**
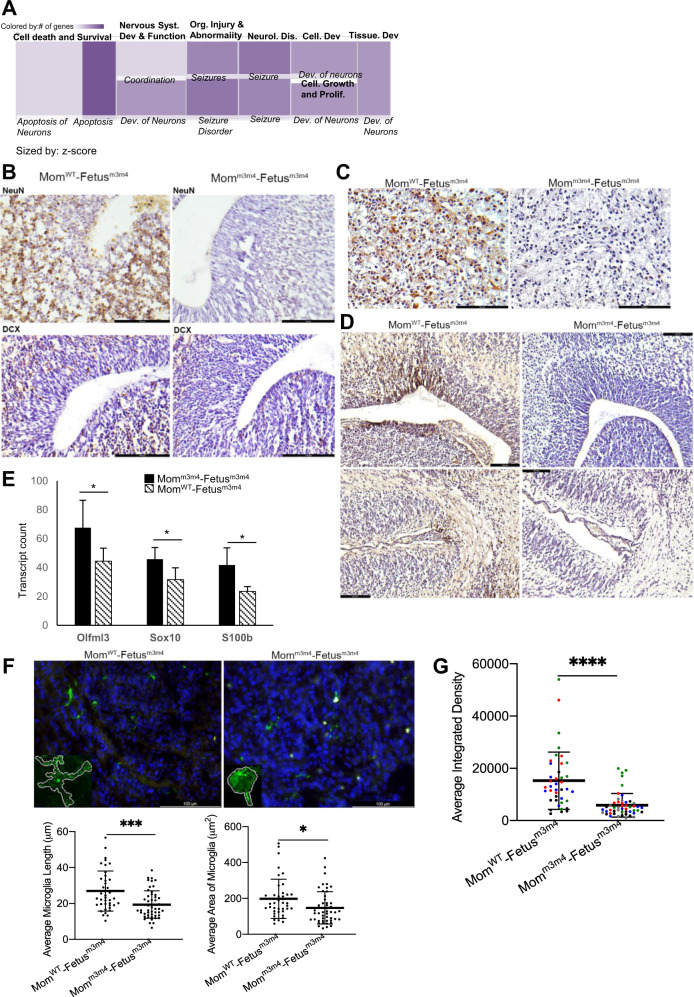
Increased neuronal loss and lack of glial cell maturation in Fetus^m3m4^ undergoing gestation in Mom^m3m4^ compared to those in Mom^WT^. **A** Ingenuity pathway analysis showing upregulation of pathways related to neuronal apoptosis and death. **B** (Upper panel) NeuN staining for mature neurons in fetal brain (representative image of *n* = 3 Mom^WT^ and *n* = 6 Mom^m3m4^). (Lower panel) Doublecortin (Dcx) staining for immature neurons in fetal brain (representative image of *n* = 4 Mom^WT^ and *n* = 5 Mom^m3m4^). **C** Serotonin staining in fetuses from Mom^m3m4^ and Mom^WT^ (representative image of *n* = 3 each). **D** IHC for GFAP + astrocytes in fetal brain (Upper panel) periventricular area and (Lower panel) cavum septum pellucidum. **E** Nanostring glial panel gene expression analysis of the fetal brain. **F** (Upper Panel) Iba1 staining of fetal brain at E17.5. Insets show traces of typically observed microglia morphology. Quantification of microglia in fetal brains (Left lower panel) length, (Right lower panel) area (*n* = 5 microglia × 2 fields from *n* = 4–5 mice). **G** Integrated density of Iba1 staining on microglia in the fetal brain. Scale bars = 100 µm. Two-tailed *t* test, *p < 0.05; ***p < 0.001.

RNA sequencing of the fetal brain showed that 10/91 (11%) differentially expressed genes were those involved in Ca^2+^ or cation channel function indicating potential neuronal pathology in Fetus^m3m4^ from Mom^m3m4^ (Supplementary Table 2). Ingenuity pathway analysis of Mom^WT^–Fetus^m3m4^ vs. Mom^m3m4^–Fetus^m3m4^ fetal brain differential transcriptomes revealed “cell death and survival”, “neuron development and apoptosis” and “seizures” as the topmost “Disease and Function” pathologies (Fig. 4A). Reduced immunohistochemistry staining for NeuN showed a significant decrease in mature neurons in the brain of Mom^m3m4^–Fetus^m3m4^ (*n* = 6) compared to Mom^WT^–Fetus^m3m4^ (*n* = 3, Fig. 4B upper panel and S3E). In addition, significantly reduced Doublecortin (Dcx, marker for immature neurons) staining in Mom^m3m4^–Fetus^m3m4^ (*n* = 5) compared to Mom^WT^–Fetus^m3m4^ (*n* = 4) suggested loss of neurons (vs. failure to mature) in pups undergoing gestation in Mom^m3m4^ consistent with our RNA-Seq findings (Fig. 4B lower panel and S3D). In correlation, serotonin staining was significantly decreased in Mom^m3m4^–Fetus^m3m4^ compared to Mom^WT^–Fetus^m3m4^ (Fig. 4C).

Since neuronal development is known to be dependent on crosstalk with astrocytes and microglia, we investigated astrocyte changes in Fetuses^m3m4^ undergoing gestation in Mom^WT^ vs. those in Mom^m3m4^. A significant reduction in GFAP + astrocytes was observed in Mom^m3m4^–Fetus^m3m4^ at E17.5 compared to Mom^WT^–Fetus^m3m4^ (*n* = 4, Fig. 4D). Analysis of glial specific RNA transcripts in E17.5 brains showed an increase in expression of S100b and Sox10 in Fetus^m3m4^ derived from Mom^m3m4^ (*n* = 4, *p* = 0.025 and *p* = 0.048, respectively), indicating a lack of astrocyte maturation vs. astrocyte loss influenced by the mother’s genotype (Fig. 4E). Analysis of microglial morphology and Iba1 expression in Mom^m3m4^–Fetus^m3m4^ showed a less ramified and more rounded morphology (mean process length 19.4 vs. 26.9 µm, *p* = 0.0003; total cell area 147.1 vs. 197.6 µm^2^, *p* = 0.017) compared to microglia in Mom^WT^–Fetus^m3m4^ (Fig. 4F). The significantly lower integrated density of Iba1 expression in addition to the rounded microglial morphology indicated a lack of microglial maturation in Mom^m3m4^–Fetus^m3m4^ (Mean ID: 15,269 vs. 5899; *p* = 0.0001; Fig. 4G).

**Fig. 5:**
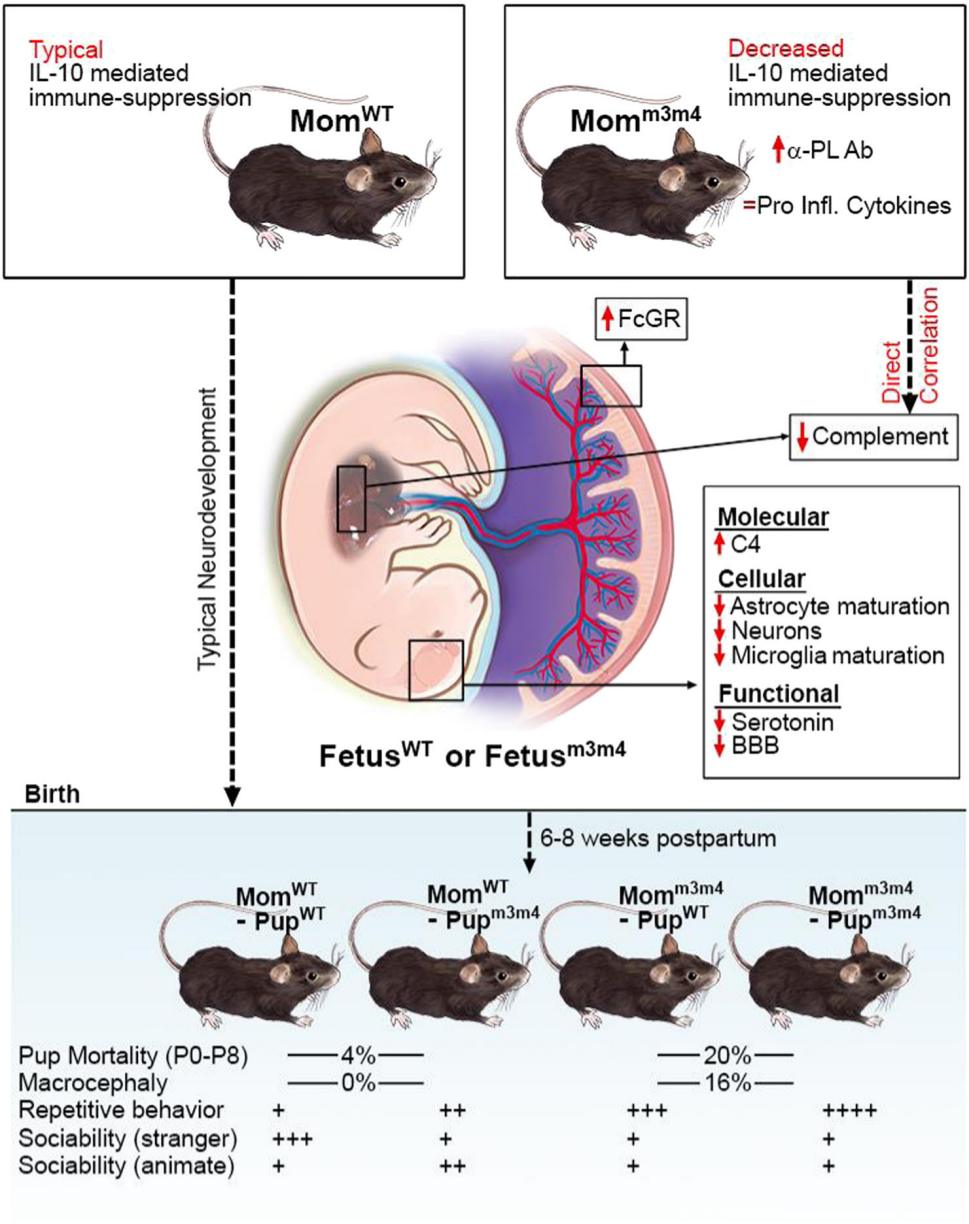
Schematic summarizing the effect of maternal genetics on fetal neurodevelopmental processes. The impact of maternal genotype on in-utero gene transcription in the offspring is evident in fetal neuropathology and postnatal ASD-like behavior.

Postnatally (P40), glial lineage-specific differential gene expression was found to cluster solely by pup’s genotype, i.e. between Pup^WT^ and Pup^m3m4^, irrespective of the mother’s genotype (Fig. S3F). This indicates that maternal influence on fetal gene transcription is restricted to the in-utero gestation period. However, such processes initiated in-utero can impact ASD-like behavior and survival postnatally (summarized in Fig. 5). Collectively our data show that maternal genetics predisposing to inflammation can lead to transcriptional and cellular aberrations in the developing fetal brain.

## Discussion

This study demonstrates that maternal genetics merely predisposing to excessive inflammation can impact transcriptional and cellular processes in the developing fetal brain in response to the mother’s gravid state. We suggest that ongoing autoimmunity or strong external inflammatory insults are not essential for eliciting harmful maternal inflammation in a genetically predisposed system. Instead, a lack of adequate immunosuppression during pregnancy, driven by maternal genotype alone, can lead to significant alterations in fetal neurodevelopment. These in-utero cellular and gene expression aberrations affected by the maternal genotype impact offspring neurobehavior and survival postnatally.

Classically, a gene–autoimmune environment interaction has been thought to be etiological for ASD, but recent developments have challenged the idea to include a double-edged nature of many immune effectors in mediating beneficial vs. detrimental effects on disease development^54^. Our data highlight the impact of maternal genetics on immunosuppression during pregnancy. We show that IL-10 is an important mediator of the crosstalk between maternal immunity during pregnancy and fetal neurodevelopment. The role of IL-10 as an essential mediator of immunosuppression in pregnancy is unclear^55–58^. However, lower IL-10/pro-inflammatory cytokine ratios have been seen in children with ASD^59^. Disruptions in IL-10-mediated anti-inflammatory processes at the materno-fetal interface have been linked to changes in adult behavior^60^. Persistently high IL-10 levels have been shown throughout pregnancy in women with systemic lupus erythematosus, possibly as an attempt to immunomodulate the existing inflammation^61^. Our findings highlight that pregnancy loss, pre-term births, higher incidence of children with ASD in women with autoimmunity or perinatal infections^17,18,22–24,62^ as well as maternal genotypes predisposing to inflammation, as shown here, may all converge on an imbalanced or inadequate state of immunosuppression during pregnancy. We show here for the first time a direct correlation between maternal IL-10 levels during pregnancy and complement expression in the fetal liver. These findings are significant because they suggest a direct influence of the maternal immunosuppressive state (vs. an inflammatory state) on fetal physiology. Complement factors have been associated with neurological diseases, albeit with variable and sometimes contrasting results in the periphery and the CNS^63–65^. Our data showing decreased complement expression in the periphery and a contrasting increase in complement in the brain of Mom^m3m4^–Pup^m3m4^, reflects similar reports on conflicting levels in the periphery vs. CNS in other neurodevelopmental disorders^66^. Further elucidation of the role of complement proteins in peripheral processes and their impact on the CNS may reveal common biology and therapeutic targets across different neurodevelopmental disorders^67^.

The concepts of “fetal programming” and “developmental origins of health and disease” suggest that the gestational environment influences early development and initiates molecular responses that impact long-term disease predisposition^33,68^. Our observations of in-utero loss of neurons, significant changes in the integrity of the BBB, glial maturation, and gene transcription based on the gestational mother’s genotype reflect early disturbances in neural induction that may impact later neuronal synapse formation and function^69^. Fetal neurodevelopmental defects seem to be reflected in postnatal phenotypes such as aberrant behavior and macrocephaly. We have previously shown that macrocephaly in Pups^m3m4^ is a combined result of increased white matter, cellular hypertrophy, and increased cell numbers^42^. Our earlier studies have shown increased neuronal caliber (hypertrophy) in Pten^m3m4^ mice^46^. The impact of transient BBB breakdown and cationic channel defects on neuronal and glial pathology in the fetus, the extent to which immaturity of microglia and astrocytes affects neuronal loss and the collective impact of all these on macrocephaly and ASD-like behavior in the adult still remain in question. Whereas gene transcription in the adult offspring brain was solely dictated by its own mutation status and not affected by the mother’s genotype (Fig. S3F for glia specific genes), preliminary IHC findings by our group suggest a reversal of fetal phenotypes in the Mom^m3m4^–Pup^m3m4^ adult. These data possibly suggest an overcompensation mechanism initiated during neurodevelopment to offset the fetal phenotype (especially neuronal) that ultimately results in a reversed adult phenotype. The cellular, protein synthesis, and apoptosis-related pathways involved in this overcompensation are still under investigation. These issues need concerted investigation and are out of the scope of the current work focused on elucidating the impact of maternal genetics on fetal neurodevelopment in ASD.

Overall, the postnatal ASD-like phenotype (behavior, pup survival, and exaggerated macrocephaly) was a reflection of both the offspring’s and maternal genotypes, whereby Mom^m3m4^–Pup^m3m4^ > Mom^m3m4^–Pup^WT^ > Mom^WT^–Pup^m3m4^ > Mom^WT^–Pup^WT^. Subtle differences in ASD-like behavior of Pups^m3m4^, whereby social behavior change from low social motivation to restricted sociability and need for sameness was dependent on the mother’s genotype, provide clues to minor differences in biological processes underlying the diverse phenotypic architecture of ASD. It is worth noting that both Moms^WT^ and Moms^m3m4^ were derived from wildtype dams and therefore are not expected to have significant behavior differences between them as shown by our data on female offspring derived from Mom^WT^ (Fig. S1B–D). Our data showing increased ASD-like phenotypes in wildtype pups derived from mothers genetically predisposed to inflammation compared to Pups^m3m4^ that are genetically predisposed but born to wildtype mothers are interesting. These findings are reflective of studies showing ASD-like behavior in wildtype offspring after induction of maternal inflammation by LPS or Poly I:C injection^30–34^. Importantly, the data presented here show induction of autism-like phenotype in non-predisposed offspring without any external stimuli and as a mere response to pregnancy in a maternal environment genetically predisposed to inflammation.

Collectively, we find that maternal genetics could be a stand-alone and important etiological factor for ASD that can influence fetal neurodevelopment and program the offspring towards an ASD-like phenotype, the severity of which is determined in conjunction with the offspring’s own genetic susceptibility. The current sample size is not sufficient to draw direct correlations of fetal aberrations with the maternal IL-10 levels since the majority of Moms^m3m4^ had low IL-10 levels. Much larger murine studies will be required to obtain sufficient Moms^m3m4^ with serum IL-10 comparable to Moms^WT^. However, our IL-10 related findings are probably the most translationally important. Further studies in this direction would involve GWA studies to identify maternal genes associated with low/high serum IL-10 levels during pregnancy in the general population as well as in women with PHTS and with autoimmune susceptibility genes. Follow-up studies on the association of maternal IL-10 levels and incidence of children with ASD in PHTS individuals or in families with a history of ASD may provide insights into possible clinical impact.

Most importantly, our study shows differences in phenotype penetrance in high-risk (genetically predisposed) offspring born to mothers that are genetically predisposed to inflammation vs. those that are not. We show that maternal genotype can impact the necessary immunosuppressive state during pregnancy and thereby modulate (increase or decrease) phenotype severity in genetically predisposed (i.e., by *Pten* mutation) high-risk offspring as well as increase neurological abnormalities in non-predisposed (*Pten* wildtype) offspring. In other words, maternal–fetal genotype interaction is important in fetal phenotype outcome and modulates severity.

## Supplementary information

Figure S1

Figure S2

Figure S3

Supplementary Table 1

Supplementary Table 2
