# Supplementary material for: Maternal genetics influences fetal neurodevelopment and postnatal autism spectrum disorder-like phenotype by modulating in-utero immunosuppression
Source: Transl Psychiatry. 2021 Jun 5;11:348. doi: 10.1038/s41398-021-01472-x (PMC8179926; doi:10.1038/s41398-021-01472-x)
Supplement: Supplementary file 1 — Figure S1 [file 41398_2021_1472_MOESM1_ESM.pptx]

## Slide 1
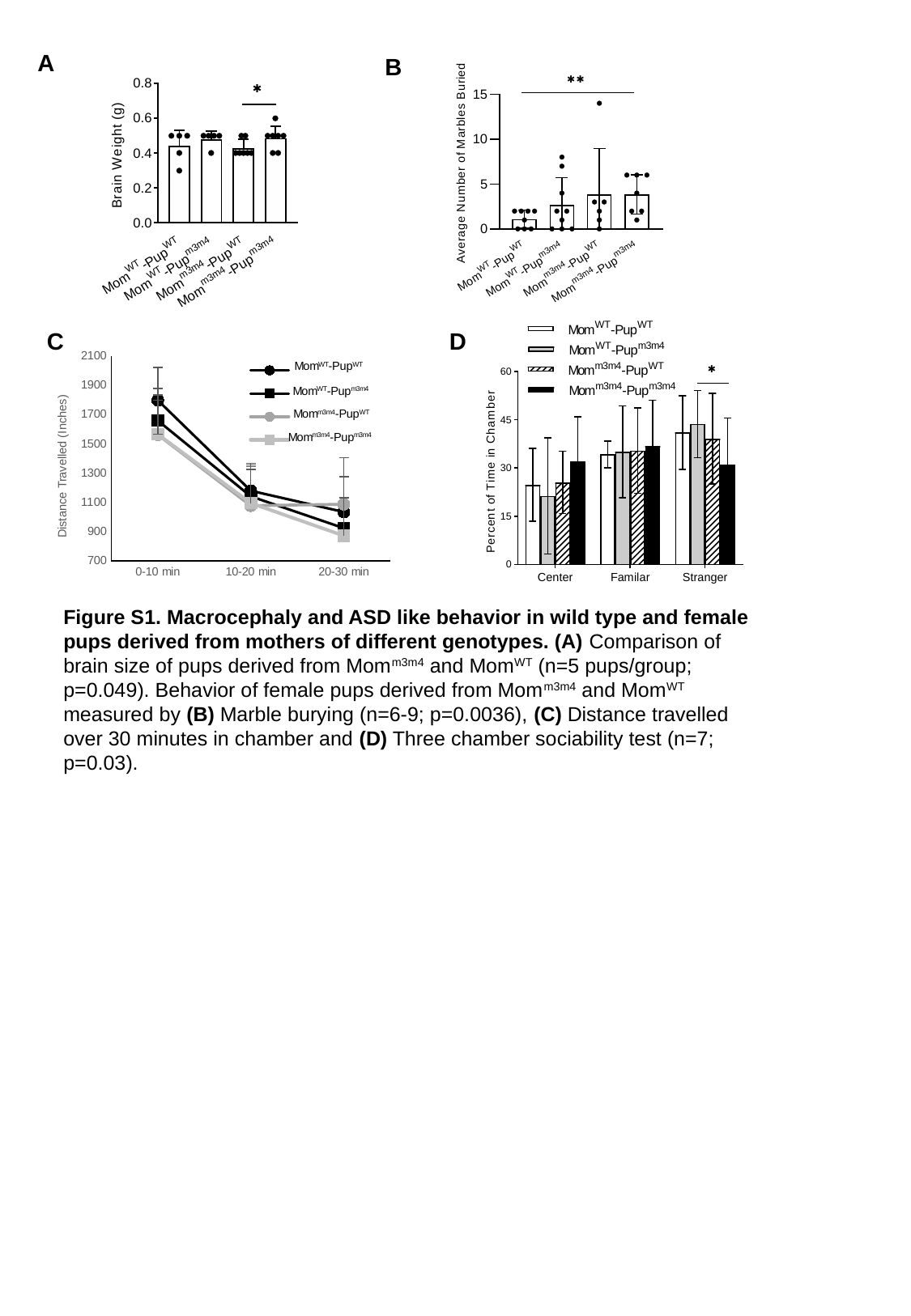

A
B
C
D
### Chart
| Category | | | | |
|---|---|---|---|---|
| 0-10 min | 1797.8075 | 1659.005666666667 | 1561.7983333333332 | 1565.886 |
| 10-20 min | 1178.63375 | 1140.294 | 1076.6425000000002 | 1094.3890000000001 |
| 20-30 min | 1033.39725 | 923.0573333333333 | 1086.5883333333334 | 870.9938 |Figure S1. Macrocephaly and ASD like behavior in wild type and female pups derived from mothers of different genotypes. (A) Comparison of brain size of pups derived from Momm3m4 and MomWT (n=5 pups/group; p=0.049). Behavior of female pups derived from Momm3m4 and MomWT measured by (B) Marble burying (n=6-9; p=0.0036), (C) Distance travelled over 30 minutes in chamber and (D) Three chamber sociability test (n=7; p=0.03).
