# Supplementary material for: Maternal genetics influences fetal neurodevelopment and postnatal autism spectrum disorder-like phenotype by modulating in-utero immunosuppression
Source: Transl Psychiatry. 2021 Jun 5;11:348. doi: 10.1038/s41398-021-01472-x (PMC8179926; doi:10.1038/s41398-021-01472-x)
Supplement: Supplementary file 2 — Figure S2 [file 41398_2021_1472_MOESM2_ESM.pptx]

## Slide 1
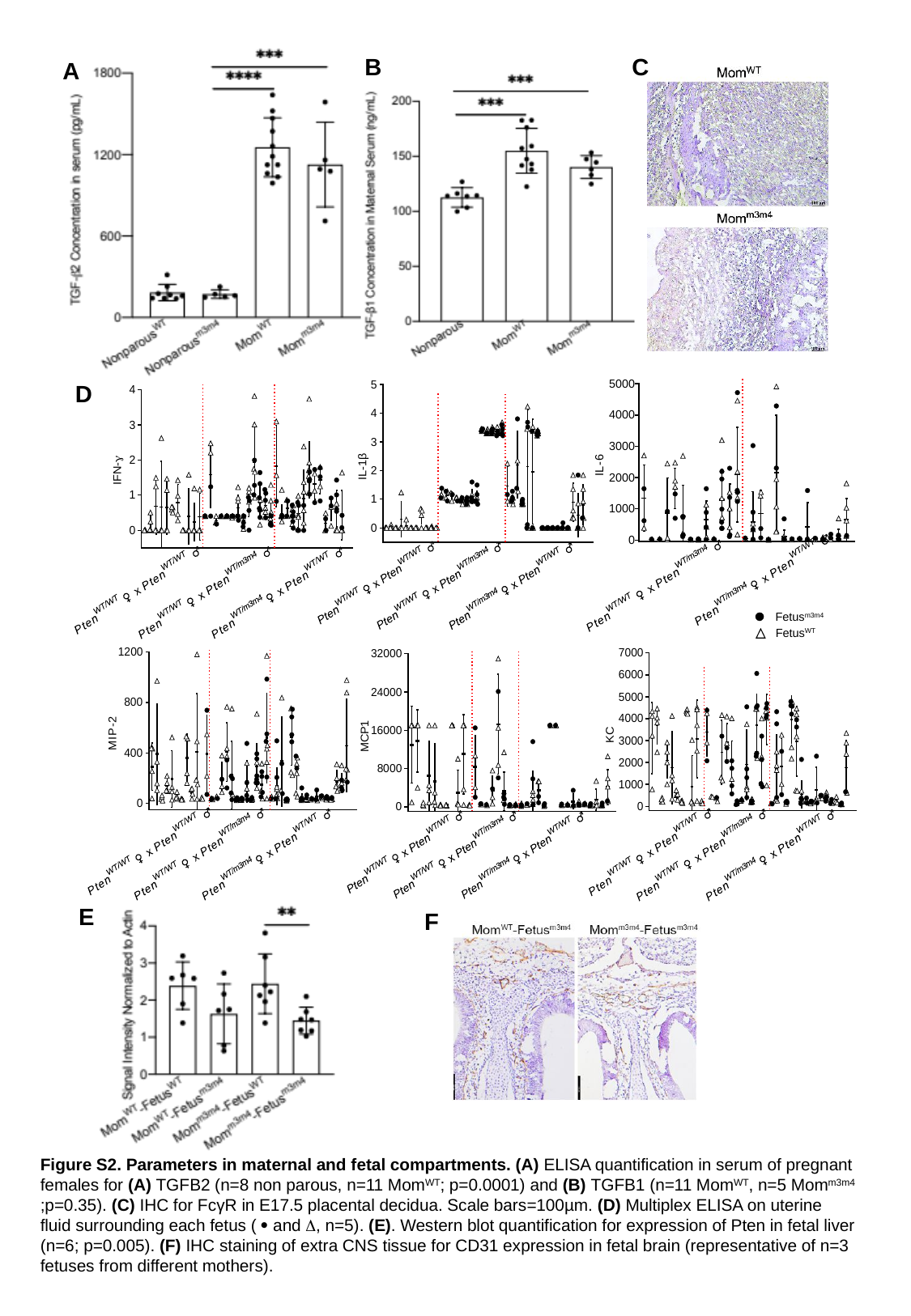

B
C
A
D
Fetusm3m4
FetusWT
E
F
Figure S2. Parameters in maternal and fetal compartments. (A) ELISA quantification in serum of pregnant females for (A) TGFB2 (n=8 non parous, n=11 MomWT; p=0.0001) and (B) TGFB1 (n=11 MomWT, n=5 Momm3m4 ;p=0.35). (C) IHC for FcγR in E17.5 placental decidua. Scale bars=100µm. (D) Multiplex ELISA on uterine fluid surrounding each fetus (  and Δ, n=5). (E). Western blot quantification for expression of Pten in fetal liver (n=6; p=0.005). (F) IHC staining of extra CNS tissue for CD31 expression in fetal brain (representative of n=3 fetuses from different mothers).
