# Supplementary material for: Maternal genetics influences fetal neurodevelopment and postnatal autism spectrum disorder-like phenotype by modulating in-utero immunosuppression
Source: Transl Psychiatry. 2021 Jun 5;11:348. doi: 10.1038/s41398-021-01472-x (PMC8179926; doi:10.1038/s41398-021-01472-x)
Supplement: Supplementary file 3 — Figure S3 [file 41398_2021_1472_MOESM3_ESM.pptx]

## Slide 1
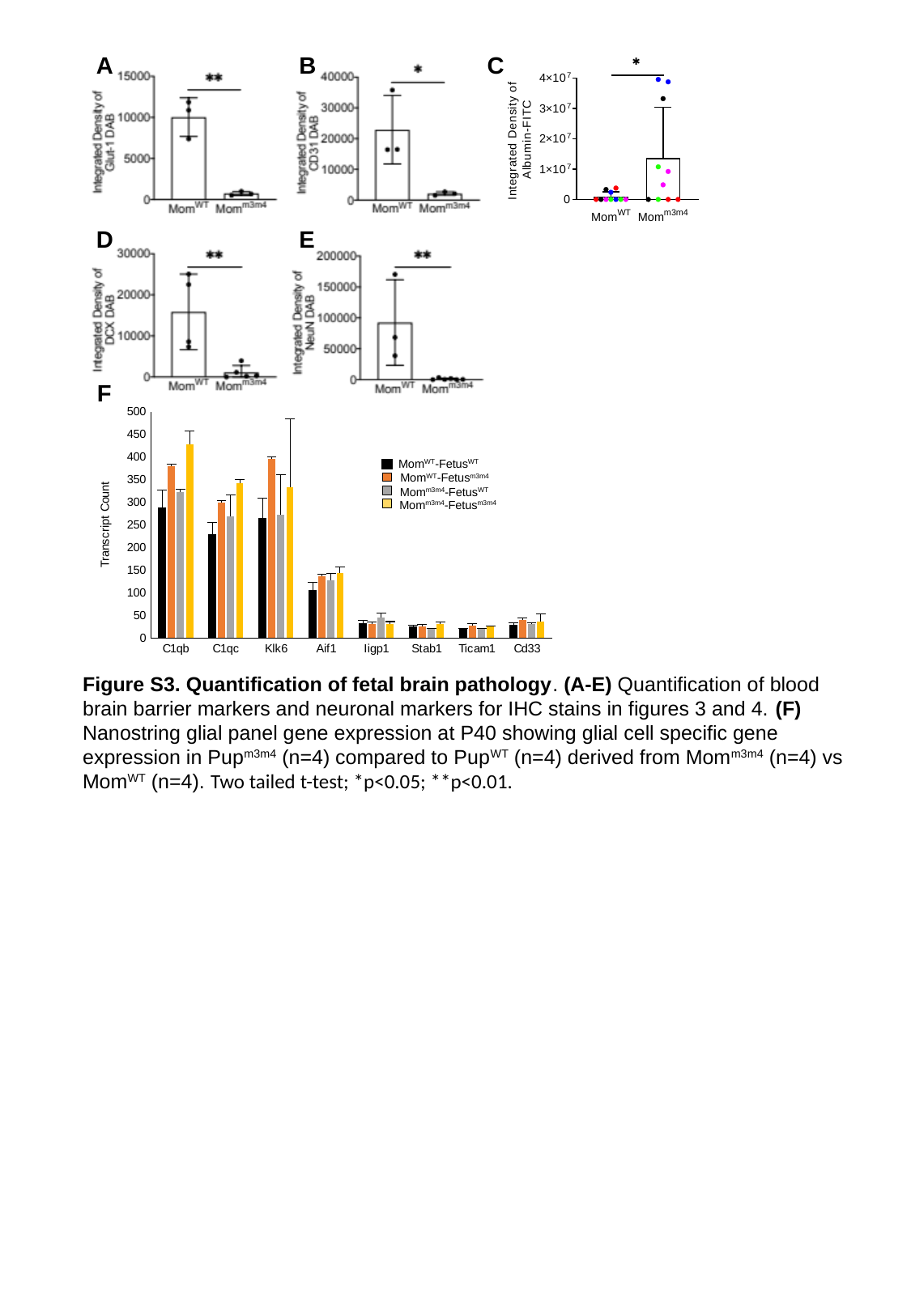

C
A
B
D
E
F
### Chart
| Category | WT-WT-P40 | WT-HET-P40 | HET-WT-P40 | HET-HET-P40 |
|---|---|---|---|---|
| C1qb | 287.18 | 379.95 | 322.95 | 427.42 |
| C1qc | 228.47 | 299.27 | 268.84 | 341.96 |
| Klk6 | 264.79 | 395.24 | 272.05 | 333.95 |
| Aif1 | 104.61 | 135.71 | 127.44 | 143.36 |
| Iigp1 | 31.83 | 30.43 | 45.56 | 30.64 |
| Stab1 | 24.3 | 25.58 | 20.4 | 30.55 |
| Ticam1 | 20.2 | 27.35 | 20.4 | 26.93 |
| Cd33 | 27.41 | 39.99 | 30.05 | 36.67 |MomWT-FetusWT
MomWT-Fetusm3m4
Momm3m4-FetusWT
Momm3m4-Fetusm3m4
Figure S3. Quantification of fetal brain pathology. (A-E) Quantification of blood brain barrier markers and neuronal markers for IHC stains in figures 3 and 4. (F) Nanostring glial panel gene expression at P40 showing glial cell specific gene expression in Pupm3m4 (n=4) compared to PupWT (n=4) derived from Momm3m4 (n=4) vs MomWT (n=4). Two tailed t-test; *p<0.05; **p<0.01.
